# Cross-cultural differences in early expectations about third party resource distribution

**DOI:** 10.1038/s41598-022-15766-7

**Published:** 2022-07-08

**Authors:** Marek Meristo, Henriette Zeidler

**Affiliations:** Department of Psychology, University of Gothenburg, Gothenburg, Sweden

**Keywords:** Psychology, Human behaviour

## Abstract

Research using non-verbal looking-time methods suggests that pre-verbal infants are able to detect inequality in third party resource allocations. However, nothing is known about the emergence of this capacity outside a very narrow Western context. We compared 12- to 20-month-old infants (*N* = 54) from one Western and two non-Western societies. Swedish infants confirmed the pattern from previous Western samples by looking longer at the unequal distribution, suggesting that they expected the resources to be distributed equally. Samburu infants looked longer at the equal distribution, suggesting an expectation of unequal distribution. The Kikuyu infants looked equally at both distributions, and did not show any specific exactions. These results suggest that expectations of equal distributions in third party allocations are affected by experience of cultural variations of distributive norms and social interaction early in development.

## Introduction

Children are highly sensitive to being treated in what they consider to be a fair way and tend to protest strongly when desirable resources are distributed unequally. Previous experimental research has demonstrated that school-aged children typically prefer egalitarian distributions, and at the end of primary school children distribute goods also according to proportional principles considering the individuals’ relative needs and merits^1,2^. Findings from several recent studies suggest that infants are able to encode distributive actions by their second year of life (e.g.,^3–5^) and possibly earlier^6–8^. In these studies, infants witness an agent distributing resources either equally or unequally between two individuals. Infants’ responses are measured via their looking times within a violation-of-expectation paradigm, i.e. infants look longer when the observed distribution deviates from their prior expectations. Based on these non-verbal studies, preverbal infants have been credited with sociomoral reasoning skills involving among others a set of evolved and adaptive principles of fairness^3,9–12^.

In a ground-breaking study, Geraci and Surian^3^ showed 16-month-olds animated movie clips depicting a fair distributor making an equal distribution and an unfair distributor making an unfair distribution of attractive resources between two puppets. The infants were then asked to choose between the pictures of the two distributors. The results showed that significantly more infants picked up the fair rather than the unfair distributor suggesting for the first time a very early rudimentary preference for fairness. In another study^4^, 19- to 21-month-old toddlers witnessed an experimenter distributing resources to two identical puppets. In one trial, the experimenter made an equal distribution, giving one object to each puppet, whereas in the second trial, the unequal scenario, the experimenter made an unequal distribution by giving both objects to one of the puppets. The toddlers looked reliably longer at the test scene after witnessing the unequal distribution of objects, suggesting that they expected the experimenter to distribute the objects equally. The authors of these studies argue for the possibility that their findings are consistent with the view that humans possess innate and evolved sociomoral norms^3,4^. These are supposed to facilitate cooperation and are affected by cultural context.

However, the details of what constitutes a fair offer are context-dependent, and in some cases, unequal divisions of resources might be more appropriate—for instance if one of the recipients is in greater need or has contributed more to the resource acquisition. Three-year old children distinguish between situations in which resources have been obtained through collaborative versus individual work^13^, and by around 5 years of age they adjust their sharing behaviors depending on the recipient^14^. Preschoolers will also take their partner's previous behavior into account^15^ and respond contingently to generous and selfish acts. Later on, children apply equity principles by giving larger amounts to partners who have worked more^16^ and from around 9 years of age, children reliably adjust their sharing behaviors to a broad range of contexts^17^. The importance of contextual influences on children's understanding of fairness has also become apparent in cross-cultural studies, which identified a large number of cultural differences—from general sharing behaviours^18,19^ to considerations of merit^20^, and friendship^21^.

In terms of distributive justice, there seems to be considerable variability in fairness principles across different communities from different parts of the world^22^. Children growing up in various places therefore observe and learn from different practices and rules about how things are distributed in everyday interactions. The fact that children are able to monitor and judge these rules from very early on seems to point towards a universal mechanism for recognizing and responding to typical patterns of social interactions.

The purpose of the current study was thus to extend the research on early fairness reasoning outside the Western perspective and test the hypothesis that infants possess a universal capacity to monitor distributive actions. From the larger perspective, the current Western culture is highly unusual and non-representative of the human species as a whole: throughout most of our evolutionary history, we lived in small-scale societies without formal laws, markets and schools; children did not grow up in small nuclear families nor were they away at school much of their time; and learning took place mostly through observation, participation, and social bonding rather than coming from conversations with adults^22^. Although research on infants’ early evaluations of resource distribution has increased during the last ten years and a number of researchers have started focusing on cross-cultural differences in older children's sense of fairness, to date no study has looked at its early development outside the typical WEIRD (i.e., Western, Educated, Industrialized, Rich, Democratic) context.

In order to fill this gap, our study included infants from two non-Western societies, Samburu and Kikuyu in Kenya, and a Western comparison group from Sweden with different levels of power distance and inequality. Power in this context is defined as control or influence over assets, rights, or individuals^23^, and measures of power distance indicate how (un)equally this influence is distributed among individual members of a society^24^. Accordingly, our samples also differ in their local Gini Index, which provides a measure for how equally resources are distributed among the members of a certain population, ranging from 0 (complete equality) to 100 (complete inequality). On the country level, Kenya's Gini Index (44.5) is much higher than Sweden's with 29.3^25^. However, Kenya also shows great variation across individual localities. Our Samburu sample was recruited in Wamba East, which has a Gini Index of 41.7; and our Kikuyu sample came from Thingithu/Tigithi Wards in Laikipia East with a Gini Index of 33.7^26^. Gothenburg on the other hand, has a local Gini Index of 24.1^27^.

The Samburu currently number around 300,000 people spread across remote parts of Northern and Central Kenya. They speak Samburu, a Nilotic language closely related to Maa, and live predominantly as semi-nomadic pastoralists^24^. Keeping livestock such as cows and goats plays an important role in the Samburu life and culture. The social organization among the Samburu is largely based on age, with leaders being significantly older than the majority of the adult population. The age-based system of rights and duties continues to be a significant feature of pastoralist societies in East Africa today, including the Samburu^28^. Gender is an equally strong factor for determining social status, with males being the undisputed heads of the family. Children are highly valued, and families typically have five or more. Infants are treated leniently until they are about 3 years of age, but as they grow older, the amount of obedience required increases. Most children start helping around the household when they are about four years old. Girls usually take care of younger siblings and help to fetch water and firewood, while boys typically look after the family's livestock. Although most Samburu today own mobile phones, communication is often restricted by lack of network or power for charging, and media access through TV or internet is usually limited to towns. Unlike other ethnic groups in Kenya, Samburu have largely upheld their cultural traditions and embraced Westernization to a much lesser degree^29^.

In order to be able to draw more general conclusions about the influence of Western-style education and values on children's developing sense of fairness, we included infants from another Kenyan group with different societal structures, the Kikuyu. Numbering around 8 million people, the Kikuyu are Kenya's largest ethnic group, living in the central part of the country^30^. Their language (Kikuyu) is part of the Bantu family. Families usually speak a mix of Kikuyu and Swahili (the national language) at home, with Swahili being more prevalent in urban areas. Traditionally, they have been small-scale farmers, cultivating maize, beans, and other vegetables and practicing animal husbandry for their subsistence. Recently, trade and wage work have become more important, and an increasing number of Kikuyu have become part of Kenya's middle or upper class, embracing business and education. While males are still considered to be the official head of the household, gender differences have rapidly decreased in recent years. Age, however, continues to be a reason for respect across Kikuyu society. Children still count as a blessing for Kikuyu, but as parents try to provide education beyond primary school, large families are becoming a financial burden. Nowadays, many families are restricting their number of children to a maximum of three. Training children to be obedient is still a major parenting goal which is instilled from infancy on. General living conditions vary vastly depending on income and range from simple wooden huts without electricity and water to huge estates. Our sample was recruited from rural areas in Laikipia East.

For comparison, we included a Western sample of Swedish infants from Gothenburg.

We hypothesized that in a strictly hierarchical society such as Samburu^29^ resources are not typically distributed equally and therefore a generalized principle of equality cannot be learned by infants through everyday observations—in contrast to a Western society such as Sweden with lower power distance and a greater emphasis on equal distributions. Consequently, we expected an opposite pattern of looking times among Samburu infants in our fairness task compared to the Swedish infants. Kikuyu infants should be somewhere in the middle, given the less pronounced social hierarchies and increasing influence of Western-style education and thinking^30^.

## Methods

The study was approved by Maseno University Ethics Review Committee (reference number: MSU/DRPI/MUERC/00616/18) and the Regional Ethical Review Board in Gothenburg Sweden (reference number: 192-18). All methods were performed in accordance with the relevant guidelines and regulations. A parent or legal guardian of the participating children provided informed consent prior to testing.

### Participants

Samburu participants included 21 infants (9 female; age range: 13–20 months; mean age: 16.4 months). Another 2 infants were tested but excluded because they did not meet the inclusion criteria (i.e., looking more than 2.5 s before looking away for more than 2.5 consecutive seconds in the test event).

The Kikuyu sample consisted of 17 infants (8 female; age range: 13–20 months; mean age: 15.2 months). Additional 3 infants were tested but excluded because they were not looking at the screen (n = 1), or did not meet the inclusion criteria mentioned above (n = 2).

Swedish participants were 16 infants (3 female; age range: 12–20 months; mean age: 16.0 months). Two infants were tested but excluded because they were not looking at the screen. Compared to the other two samples, the Swedish sample had slightly more male than female participants. However, given that previous research has found no evidence of interactions between gender and fairness considerations in infants^4,8^, we do not believe that the slightly different sample composition had any influence on our results.

The sample size was specified on the basis of the effect size from Meristo and Surian^6^ which examined infants’ reasoning about resource distributions in the context of identical animated events using the violation-of-expectation method and a 2 × 2 between-subject design. The condition x event effect size (*η*_*p*_^2^) in their study was 0.17. An a priori power analysis using G*Power, based on this previous effect size, suggested that 80% power at the *α* level of *p* = 0.05 required a minimum number of eight participants per cell for a 3 × 2 design. The post hoc analysis using G*Power revealed an achieved power of 84% based on the effects size of the current 2 × 3 ANOVA of *η*_*p*_^2^ = 0.184.

Participants for the Samburu part of the study were recruited from rural settlements (manyattas) in Wamba East. The Kikuyu families were recruited from rural households in Thingithu and Tigithi wards in Laikipia East. In both places, local assistants contacted families with children in the desired age range and informed them about the research program. Parents who were willing to participate were listed and later contacted again to confirm the location and dates of the study. All testing took place at local primary schools. Upon arrival, parents received written information about the procedure in the country's official languages (English and Swahili) and were encouraged to ask any questions they might have. Whenever necessary, local assistants helped with translations into the local languages (Samburu and Kikuyu).

The Swedish sample was collected from Gothenburg.

### Materials and procedure

In the fairness task^7^ infants were randomly assigned to an equal and unequal condition. Each condition consists of a familiarization phase and a test phase. In the familiarization phase, infants are shown two yellow triangles, with eyes and a mouth. This phase is aimed at introducing the two self-moving agents who will be given strawberries in the following test phase.

**Figure 1:**
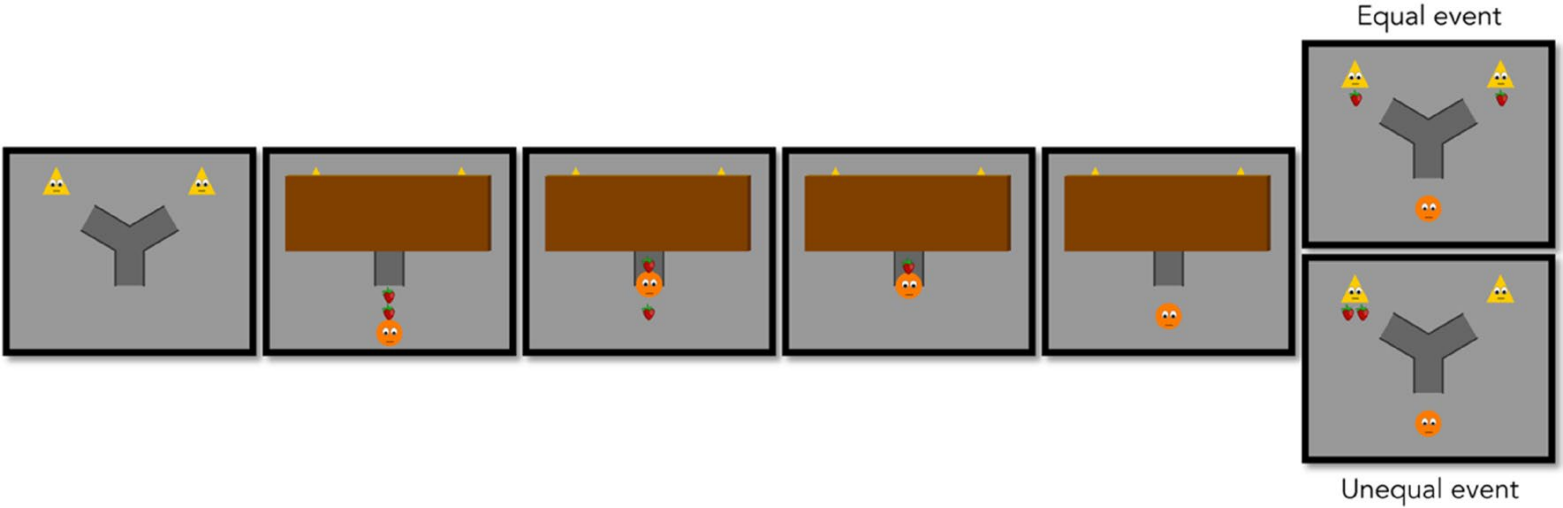
Selected frames from the test phase of the fairness task.

The test phase starts with the two triangles in place on the upper part of the screen, and below them a Y-shaped path leading to each of them (see Fig. 1 and supplementary material). Next, an occluder is lowered and then a distributor (an orange circle with eyes and a mouth) enters the scene carrying two strawberries. The distributor then twice enters the path from below, each time carrying one strawberry and placing it behind the occluder. When the distributor has delivered both strawberries, the occluder is removed revealing one strawberry in front of each triangle in the equal event, or both strawberries in front of one of the two triangles in the unequal event. The whole sequence lasts for 17 s, after which the animation freezes and infants will see the paused scene until the end of the test phase (i.e., until they look away from the screen for more than 2.5 consecutive seconds, the criterion used in previous studies^7^. Infants’ looking times were measured from the moment when the strawberries became visible from behind the occluder until the end of the test phase. According to the violation of expectation paradigm, infants will look longer at the unequal compared to the equal distribution if they expect the strawberries to be distributed equally (consistent with previous findings in 7). And in reverse, infants will look longer at the equal distribution if they expect an unequal one.

Infants’ looking was recorded with Tobii Pro Nano (Tobii Technology, Sweden) near infrared eye tracker at 60 Hz, and the results were analyzed using Tobii Pro Lab software. At all field sites, infants were seated on a parent’s lap approximately 50–70 cm from a 17-inch laptop screen used to display the stimuli. The parents were instructed not to interfere or communicate with their infants during the test session.

## Results

**Figure 2:**
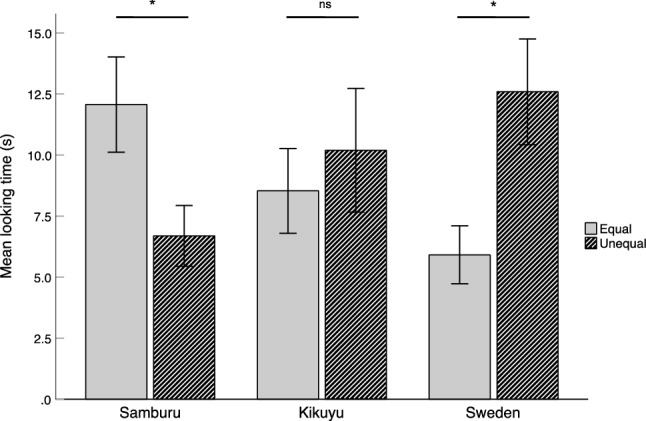
Mean looking times to the equal and unequal distribution conditions for each group and condition. Error bars show standard errors of the mean. Asterisks denote significant differences between conditions (*p* < 0.05, two-tailed).

Infants’ looking times in the test phase were analyzed using an ANOVA with Society (Samburu vs. Kikuyu vs. Sweden) and Distribution event (Equal vs. Unequal) as between-subject factors. There was a significant Society x Distribution interaction effect *F*(2, 48) = 5.33, *p* = 0.008, *η*_*p*_^2^ = 0.182. There were no main effects for Society (*p* = 0.997) or Distribution (*p* = 0.526). The Samburu infants looked significantly longer at the Equal than the Unequal event, *t*(19) = 2.27, *p* = 0.035, *d* = 1.01, the Kikuyu infants looked about equally at both events *t*(15) = 0.53, *p* = 0.604, *d* = 0.26, and the Swedish infants spent less time looking at the Equal compared to the Unequal event, *t*(14) = 2.71, *p* = 0.017, *d* = 1.35 (see Fig. 2).

We also examined infants’ general looking pattern during the test event from the beginning to the end of the movie. In each sample, there was no significant difference in total looking of the infants who saw the equal or unequal distribution event, *p*_Samburu_ = 0.915, *p*_Kikuyu_ = 0.416 and *p*_Swedish_ = 0.974. An ANOVA with Society (Samburu vs. Kikuyu vs. Sweden) and Distribution event (Equal vs. Unequal) as between subject factors and the looking at the total test event as the dependent variable revealed no significant effects, all *F*s < 1.19, all *p*s > 0.31. Thus, infants from all three populations were equally attentive during the whole test event and followed the distribution event carefully from the beginning.

## Discussion

Our results demonstrate that infants from diverse cultural backgrounds differ in their reactions to resource distributions from early on. We employed an established non-verbal violation-of-expectation looking time paradigm to measure infants’ attention to equal and unequal distributive actions. Previous research has found that 7- to 21-month-old infants from Western populations consistently look significantly longer at unequal distributive events, demonstrating an expectation of equal distribution of resources among identical individuals^4–8^. Our current findings from non-Western populations complement the general Western pattern and demonstrate cross-cultural variation in children's expectations of resource distributions. While Swedish 12- to 20-month-old infants in our study performed similarly to previous Western samples, Samburu infants demonstrated the opposite pattern, expecting unequal distributions and looking significantly longer when the resources were distributed equally. Kikuyu infants on the other hand did not seem to hold specific expectations and looked about equally in both test conditions. The cross-cultural variation found in our study suggests that notions of equality in third party resource allocations are dependent on cultural groups.

The animation used in the current study captures a most basic and simple distributive situation between anonymous individuals without any context of their previous or later interactions. In a typical large-scale society such as Sweden, distributive judgements are often made without taking personal experiences or possible future interactions between specific individuals into account, making it more important to apply generalized, abstract rules. In a small-scale society such as Samburu, on the other hand, people seldom interact with total strangers, and most distributive actions involve long-term personal relationships^28^. While it seems more important to balance resources immediately among strangers who might never interact again, distributions in small-scale settings might be equaled out across much longer periods of time. Samburu—and to some extent also Kikuyu—children might thus more often witness what appears to be an unequal distributive action, since they are not (yet) keeping track of the complex long-term relationships between individuals. Several studies involving older Samburu and Kikuyu children have indeed show that they neither distribute resources evenly among each other, nor think that equal distributions are a general cultural norm^20,31^—findings which have been substantiated by research from various other small-scale communities (e.g.,^18,19^).

There might be of course alternative explanations for the cultural differences in the looking time task used in our study based on attentional-perceptual biases. After all, infants in rural Kenya are much less used to looking at screens and following animated movies than their Swedish peers. However, infants from all three samples were equally attentive throughout our eye tracking task suggesting that differences in our dependent measure cannot be attributed to differences in everyday experiences of screen-based animations. Our study is thus among the first to demonstrate that portable eye tracking can be successfully applied for in studies with non-Western infants in remote field sites, opening up a much-needed avenue for a more global research program.

However, there are other limitations to our study. First, the current conclusions are based on a single looking-time measure and small samples from a limited number of populations. Future research should therefore be extended to include a more diverse set of non-Western populations and confirm our findings in wide range of looking-time tasks. Second, our looking-time results would be even more robust if they were accompanied by measures from behavioral experiments. There is currently no experimental evidence from cross-cultural studies available about the universality of the violation-of-expectation looking time paradigm. Even within the Western samples, infants sometimes look longer to the expected compared to the unexpected outcome, if the task is challenging (e.g.,^32^). Therefore, the finding that infants look longer at the equal outcome in the Samburu sample needs further independent confirmation. Future research should ensure that the differences in the three samples were indeed differences in infants’ expectations of resource distributions and not differences in how difficult the task was for infants. Yet, the results in the current study do already confirm that infants from all three populations showed similar overall looking patterns, suggesting no systematic cultural differences in the level of difficulty of our eye tracking task in general. Third, direct observations of local distributive practices and detailed infant-caregiver conversations would provide a more substantial backdrop and help us to unravel the origins of the observed cultural differences. Fourth, the age range of the participants in the current study (i.e., 12–20 months) was larger than the one typically used in studies with Western samples. These studies have shown specific expectations of fairness already at 9–10 months of age, and using simple distributions (of two items), even younger infants demonstrate an expectation for equal distributions^6–8^. It is possible that the age group used in this study is too old to reveal the initial state of infants’ fairness expectations in the non-Western cultures. However, our samples suggest that by the age of 20-months infants’ notions of equality in distributions seem to be affected by their cultural surroundings.

Overall, our findings have revealed early differences in distributive expectations among infants growing up in diverse societies. While it is important to be cautious when drawing conclusions from single study with limited samples and one single dependent measure, the current study suggests that the foundational principles of human social cognition are affected by culture-specific experiences. Our research with non-Western infants provides a much-needed broader perspective on early normative development and thus lays the foundation for future work on human social cognition and morality.

## Supplementary Information

Supplementary Information.

## Data Availability

All data analysed during this study are included in this published article and its supplementary information files.
